# Objective, long-term mobility data in patients with chronic pain after lumbar spine surgery treated with spinal cord stimulation—a prospective, multicenter trial

**DOI:** 10.1136/rapm-2025-106726

**Published:** 2025-07-10

**Authors:** Tobias Hallén, Kaare Meier, Jan Willem Kallewaard, Bart Billet, Lars Elzinga, Johannes Schapendonk, Eric Van den Bosch, Xander Zuidema, Kristin Lilja Eyglóardóttir, Helga Gulisano, Kliment Gatzinsky

**Affiliations:** 1 Department of Neurosurgery, Sahlgrenska University Hospital, Gothenburg, Sweden; 2 Department of Clinical Neuroscience, Institute of Neuroscience and Physiology, Sahlgrenska Academy, University of Gothenburg, Gothenburg, Sweden; 3 Department of Neurosurgery, Aarhus University Hospital, Aarhus, Denmark; 4 Department of Anesthesiology, Aarhus University Hospital, Aarhus, Denmark; 5 Department of Pain Management, Rijnstate Hospital, Arnhem, The Netherlands; 6 Pain Department, Amsterdam University Medical Centres, Amsterdam, The Netherlands; 7 Department of Anesthesiology, AZ Delta, Roeselare, Belgium; 8 Department of Anesthesiology, Bravis Hospital, Roosendaal, The Netherlands; 9 Department of Anesthesiology, Diaconessenhuis Hospital, Zeist, The Netherlands; 10 Department of Anesthesiology, MCL Leeuwarden, Netherlands, Leeuwarden, The Netherlands; 11 Department of Neurosurgery, Aalborg University Hospital, Aalborg, Denmark

**Keywords:** Spinal Cord Stimulation, CHRONIC PAIN, Pain Management

## Abstract

**ABSTRACT:**

**Objective:**

Spinal cord stimulation (SCS) is a treatment option for chronic neuropathic pain conditions when conventional therapies have failed. However, objective and measurable long-term data on the effects of SCS are lacking. This study evaluates changes in objectively recorded mobility and the correlation with patient-reported outcomes in SCS-treated patients with intractable back and/or leg pain following lumbar spine surgery.

**Methods:**

Fifty patients were enrolled. Baseline mobility was recorded over 4 weeks using an external neurostimulator with a built-in accelerometer. Patients achieving ≥50% pain relief during a subsequent SCS test trial received a permanently implanted stimulator at the same site. Mobility data were extracted at 3, 6, and 12 months and compared with baseline alongside work status, medication usage, and patient-reported outcome measures.

**Results:**

Thirty-seven patients received a permanent stimulator, and 32 completed the 12 month follow-up of activity data. Time spent in mobility episodes lasting at least 30 s was 34±27 min/24 hours for SCS-patients at baseline compared with 92±51 min for healthy controls (p=0.009). Mobility increased gradually after SCS, reaching statistical significance at 12 months (p=0.045). The increase was significant in patients with predominant leg pain (35±27 to 54±32 min, p=0.011) but not in those with predominant back pain. Significant improvements were observed in working ability, medication reduction, and self-reported outcomes. Increased mobility correlated significantly with reduction in self-reported disability (p=0.044) and leg pain (p=0.046).

**Conclusion:**

The new objective data indicate that SCS has beneficial, long-term effects on mobility in patients with intractable leg pain after spine surgery.

**Trial registration number:**

NCT04710355.

**WHAT IS ALREADY KNOWN ON THIS TOPIC**

- Spinal cord stimulation (SCS) is an established treatment for chronic neuropathic pain, but most studies rely on subjective, patient-reported outcomes. Long-term objective data on the effectiveness of SCS are needed.

**WHAT THIS STUDY ADDS**

- This study provides the first long-term, objectively recorded data on changes in mobility during SCS treatment, showing significant improvements in patients with chronic intractable leg pain after lumbar spine surgery. Correlations were demonstrated between increased mobility and reduction in self-reported disability and leg pain.

**HOW THIS STUDY MIGHT AFFECT RESEARCH, PRACTICE OR POLICY**

- The study presents a method for objective, long-term assessment of SCS effects on mobility and highlights the value of integrating objective measures when evaluating the overall effects of SCS on chronic pain in research and clinical practice.

## Introduction

 Spinal cord stimulation (SCS) is an established treatment option for patients with chronic neuropathic pain who have not responded sufficiently to other treatments.^1^ However, in recent years, the robustness of the evidence regarding the effects of SCS has been questioned.^2–5^ Importantly, when accounting for the effects of SCS, the outcomes are based on self-reporting, standardized questionnaires which are inherently subjective and may not align with objective data. Hence, objective, quantifiable data on the effectiveness of SCS for treatment of chronic pain in the long term (≥12 months)^4^ are warranted.^3^

A correlation between pain reduction and improvement in physical activity has been indicated in patients treated with SCS, using accelerometry.^6 7^ However, only short-term data are available with up to 1 week of non-continuous registration. New technology enables objective assessment of mobility in patients treated with SCS over longer periods, using a stimulator that, besides delivering stimulation, also registers and stores position and mobility data.^8–10^ In this study, we use this accelerometry-based technology to assess mobility in SCS-treated patients with chronic, intractable back and/or leg pain after lumbar spine surgery, a condition referred to as persistent spinal pain syndrome type 2 (PSPS2).^11^ Physical activity levels are low among these patients, which might impact general health.^12^ We also investigate the correlation between the objective mobility data and subjective, patient-reported outcome measures (PROMs) commonly used to evaluate effectiveness in pain treatment.

## Methods

### Study design

This investigator-initiated, non-industry sponsored, prospective, multicenter trial included patients from seven hospitals in Belgium, Denmark, Netherlands, and Sweden.

Data were collected and managed using the Research Electronic Data Capture (REDCap) tool (Vanderbilt University, Nashville, TN, USA), a secure, web-based, software platform designed for research studies. All enrolled patients provided a signed informed consent before study inclusion. Study participants were assigned an identifier code unrelated to personal information. Inclusion criteria were age 18–70 years, therapy-resistant back and/or leg pain for at least 6 months after previous lumbar spine surgery, with an average pain score ≥50 on a scale of 0 (no pain) to 100 (worst pain imaginable) using the Visual Analog Scale (VAS). Exclusion criteria were inability to understand the treatment or the stimulator, active addiction to drugs, alcohol, and/or medication, psychiatric disease affecting the evaluation, local infection/other skin disorder at the site of surgery, pregnancy, coagulation disorders, and active malignant conditions (online supplemental file 1).

### Procedures

Mobility was recorded using the Intellis neurostimulator (Medtronic, Minneapolis, MN, USA) equipped with integrated, accelerometer-based technology (AdaptiveStim Diary, Medtronic) that continuously registers and stores data on seven different body positions: upright (standing or sitting straight), upright mobile, reclining (half-sitting) and four lying positions, allowing long-term, objective monitoring of positions and movement. The software provides summary reports showing the percentage of time spent in each position. Recordings were performed over 4 weeks to determine baseline parameters using an external Intellis device adhered to the skin of the upper buttock. For baseline comparison, five healthy individuals (48.4±18.8 years vs 54.5±11.5 years for SCS-treated patients, p=0.32) without pain conditions were recruited.

After baseline registration, the external device was removed, and patients were implanted with one or two eight-contact SCS leads (Vectris, Medtronic) according to standard practice. Percutaneous technique with insertion of leads into the dorsal epidural space of the spinal column was utilized. Intraoperative stimulation evoking paresthesia optimized lead placement, ensuring ≥80% coverage of the painful area. After final placement and subsequent fixation of the lead(s) to the thoracolumbar fascia and connection to an extension lead, a 2 week test trial followed. Stimulation modes included conventional, paresthesia-based (tonic) stimulation, and paresthesia-free high dose/density (HD)^13^ and differential target multiplexed (DTM)^14^ stimulation. In patients achieving ≥50% pain relief during the test trial, the extension was removed, and the lead(s) were connected to a permanent stimulator placed in the subcutaneous fat at the same site as the external stimulator used for baseline recordings. The AdaptiveStim unit was activated for continuous recording of body positions and mobility. Data were extracted at 3 months (recordings from 2 weeks until 3 months), 6 months (3–6 months), and 12 months (6–12 months) after implantation.

### Outcomes

#### Primary outcome

##### Mobility

Change from baseline in mobility (upright mobile position) at the 12 month follow-up, expressed as percentage per 24 hours. One percent corresponds to 14.4 min of mobility per day. Recordings started after 30 s in upright mobile position to ensure registration of longer, continuous activity periods (eg, walking).

### Secondary outcomes

#### Pain

Changes from baseline in self-reported pain using VAS (score 0–100) for current pain, the least pain last week, and the worst pain last week, in the back and leg, respectively. To determine the predominant type of pain at baseline, a composite measure including all three pain scores was used. The minimal clinically important difference (MCID) for VAS in chronic pain is 20%.^15^

#### Functional disability

Change from baseline in self-reported Oswestry Disability Index (ODI). The ODI assesses pain-related disability in patients with low back pain.^16^ It describes functional aspects of daily living, including 10 items. A total score is presented in percentage ranging from 0% (no disability) to 100% (maximum disability). MCID is 10 points.^17^

#### Health-related quality of life (HRQL)

Change from baseline in self-rated HRQL using the VAS of the EuroQoL group five dimensions (EQ-5D) questionnaire.^18^ The scale ranges from 0 (worst health) to 100 (best possible health). MCID for EQ VAS for patients operated for disk herniation or spinal stenosis is 12 points.^19^

#### Sleep

Change from baseline in self-reported sleep quality using the Pain and Sleep Questionnaire Three-Item Index (PSQ-3). Although originally comprising eight questions, combining PSQ 1, 4, and 5 (trouble falling asleep, awakened by pain during night, and awakened by pain in the morning) has been shown to adequately assess the impact of chronic pain on sleep.^20^ Additionally, stimulator data were extracted hour-by-hour between 10 pm and 7 am to analyze time spent in lying positions and the number of position changes during the night. These data were available for a 4 week period prior to each data extraction at baseline and at the 3-, 6-, and 12 month follow-ups.

#### Work status

Change from baseline in working ability (the percentage of full time a patient was working). Patients were divided into two groups based on baseline data: pensioners (including disability pensioners who were unfit for work), and patients able to work full-time or part-time, or on sick leave.

#### Medicine consumption

Change from baseline in pain-related medicine usage. Medicines were categorized into four groups: opioids, antidepressants, anticonvulsants, and sleep medication (benzodiazepines, antihistamines, sedatives).

#### Correlation analysis

Correlations between stimulator-derived mobility data and self-reported outcomes were analyzed. Additionally, analysis was performed of the correlation between PSQ-3 scores and objectively recorded night data of time spent in lying positions and number of position changes, respectively.

#### Adverse events

The number and type of adverse events were documented for all patients (n=50) who provided informed consent.

### Statistical analysis

#### Power analysis

The power analysis was based on a paired-samples t-test of the difference in VAS scores, as no long-term data on objectively recorded mobility after SCS treatment are available. To detect a two-point difference in mean VAS between baseline and 12 months postimplantation, assuming an SD of 3, 90% power and 5% alpha, with expected rates of 30% for loss to follow-up or unsuccessful test trial, we aimed to recruit a minimum of 40 patients.

Descriptive data are presented as numbers with percentages and means with SD. Due to skewed data distribution, the Wilcoxon signed rank test was used to compare scores before and after SCS treatment. Spearman’s correlation coefficient was applied for correlation analyses. All tests were two-sided, with p<0.05 considered significant. All statistical calculations were performed using SPSS, V.29 (IBM, Armonk, NY, USA). Data were visualized using GraphPad Prism, V.10 (GraphPad Software, Boston, MA, USA).

## Results

Patient inclusion started in January 2021 and ended in February 2023. Due to the ongoing Covid pandemic, a higher risk of patient drop-out was anticipated. Therefore, before study start, we decided to include 10 additional patients beyond the 40 required by the power analysis. Of the 50 enrolled patients, 48 completed baseline registration with an external stimulator, and 45 underwent lead implantation for a 2 week test trial (figure 1). Thirty-seven patients had a stimulator implanted, of which two were excluded due to new-onset debilitating arthrosis pain in knee and hip, respectively, affecting mobility. One patient withdrew due to the need for multiple SCS revision surgeries. In total, 34 (92%) of the implanted patients completed the study questionnaires at baseline and at 12 months, of which 32 (86%) had objective data from the AdaptiveStim Diary. For two patients, data could not be extracted due to technical issues (figure 1). Baseline characteristics for the 34 included patients are shown in table 1.

**Figure 1:**
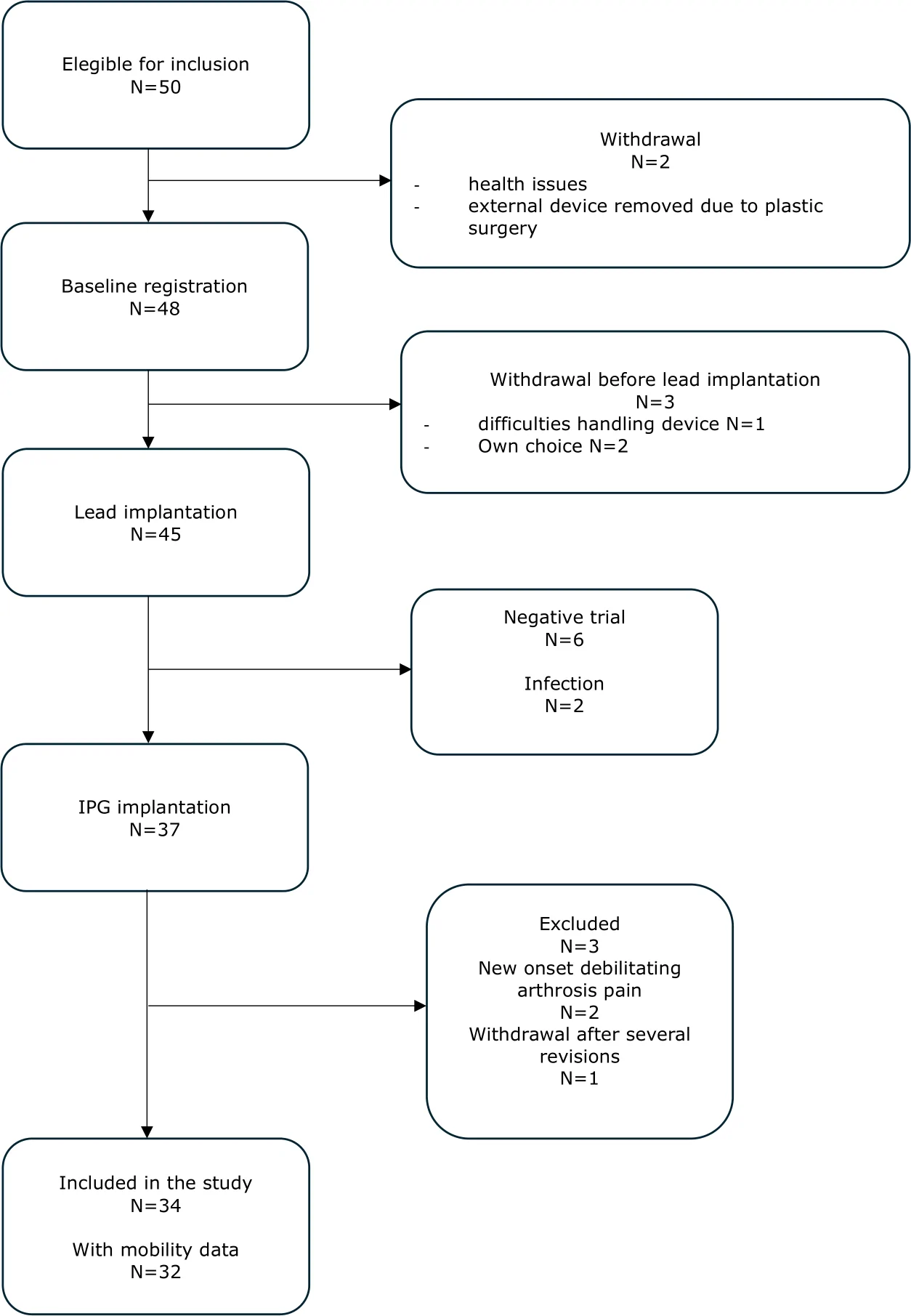
Flow chart of the included patients.

**Table 1:** Patient characteristics

Age, y (SD)	54.5±11.5
Gender, N (%)	
Male	16 (47)
Female	18 (53)
Previous back surgeries, N (%)	
1	14 (41)
2	10 (29)
3	8 (24)
4	1 (3)
5	1 (3)
Dominating pain, N (%)	
Back	12 (35)
Leg	19 (56)
Same	3 (9)
Number of leads, N (%)	
One	19 (56)
Two	15 (44)
Number of Med groups, N (%)	
Zero	8 (24)
One	9 (26)
Two	13 (38)
Three	4 (12)
Four	0 (0)
Working status, N (%)	
Able to work	18 (53)
Unable to work (pensioner, sickness benefit, unfit)	16 (47)
Stimulation mode, N (%)	
Tonic	9 (28)
HD	15 (47)
DTM	8 (25)

DTM, differential target multiplexed; HD, high density/dose.

### Primary outcome

#### Mobility

At baseline, time spent in mobility episodes lasting at least 30 s was 2.4±1.9% compared with 6.4±3.5% in healthy controls (n=5), p=0.009, corresponding to 34±27 and 92±51 min per 24 hours. At 12 months, mobility increased by 30% for the whole group of 32 patients, reaching statistical significance (2.4±1.9% at baseline vs 3.1±2.1% at 12 months, p=0.045, figure 2A). This corresponds to an increase from 34±27 to 44±31 min daily.

**Figure 2:**
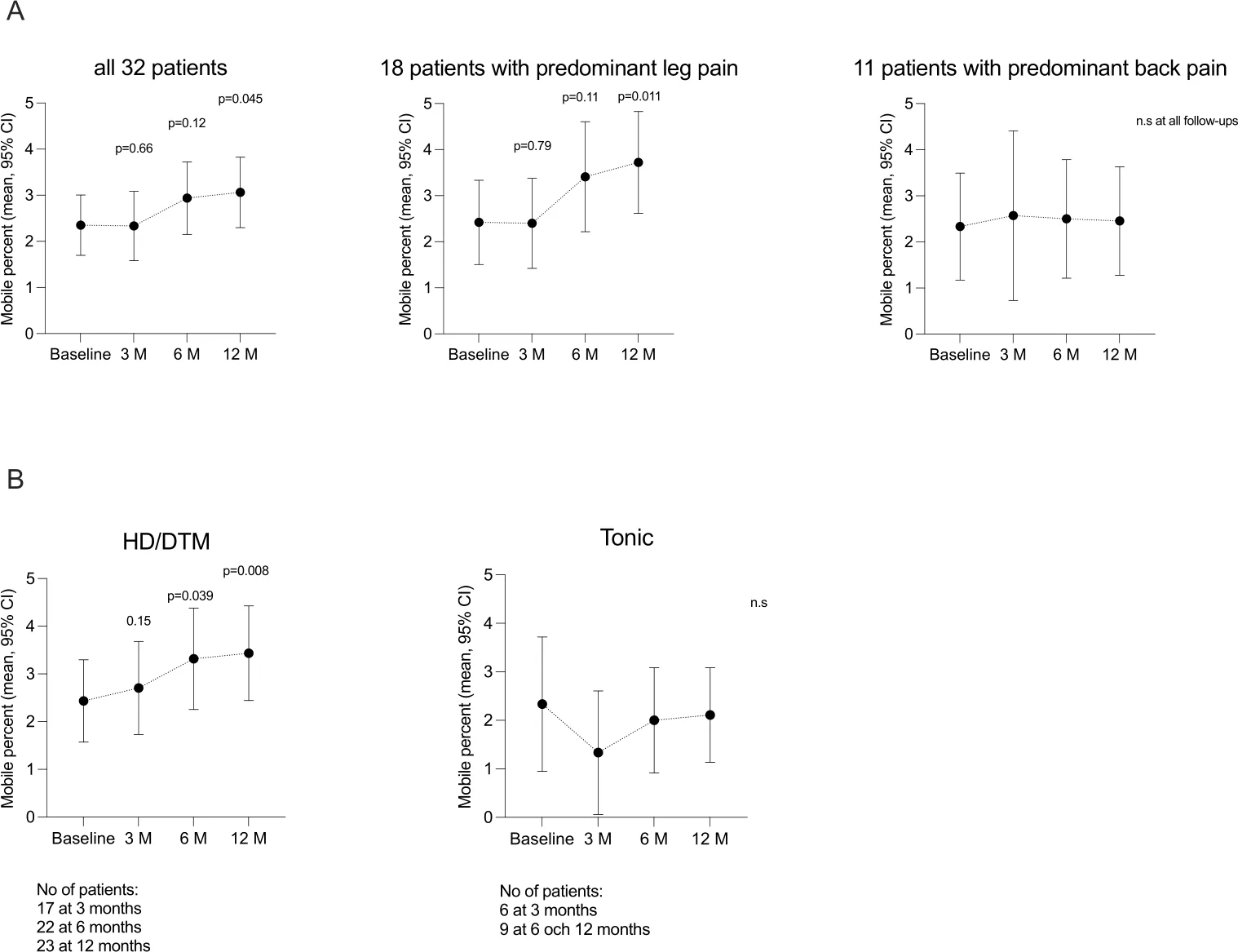
**(A**) Change in mobility (%) between baseline and follow-ups for all included patients with mobility data (n=32), as well as for those with predominant leg pain or back pain, respectively. Three patients scored equally for leg and back pain and are not included in the graphs for predominant leg and back pain. (B) Change in mobility (%) between baseline and follow-up for patients treated with HD/DTM or tonic stimulation, respectively. HD, high density/dose; DTM, differential target multiplexed.

Subgroup analyses showed a significant increase in mobility in patients with predominant leg pain (2.4±1.9% to 3.7±2.2%, corresponding to an increase from 35±27 to 54±32 min, p=0.011), but not in those with predominant back pain (2.3±1.8% to 2.5±1.8%, p=0.74, figure 2A). No significant changes were observed in upright and reclining positions (data not shown). Nine, 15, and 8 patients used tonic, HD, and DTM stimulation, respectively. The proportion using tonic stimulation was 28% in the group with predominating leg pain, and 27% in the group with predominating back pain. Paresthesia-free modes showed better outcomes regarding increase in mobility at 12 months, compared with tonic stimulation (p=0.008 and p=0.59, respectively, figure 2B).

### Secondary outcomes

#### Patient-reported outcome measures

VAS, ODI, and EQ-5D VAS scores all improved significantly at 12 months (figure 3A). Mean VAS scores for worst back pain decreased from 75.7±22.6 to 40.9±28.5 (p<0.001), and for worst leg pain from 80.7±15.9 to 42.1±26.1 (p<0.001). No significant differences in the magnitude of leg or back pain reduction were observed between paresthesia-free and tonic stimulation (data not shown). ODI scores decreased from 52.3%±13.6% at baseline to 31.9%±16.7% (p<0.001). EQ-5D VAS increased from 52.9±18.7 to 66.4±18.0, p=0.001. The outcomes exceeded the MCID for the respective PROMs.^15 17 19^

**Figure 3:**
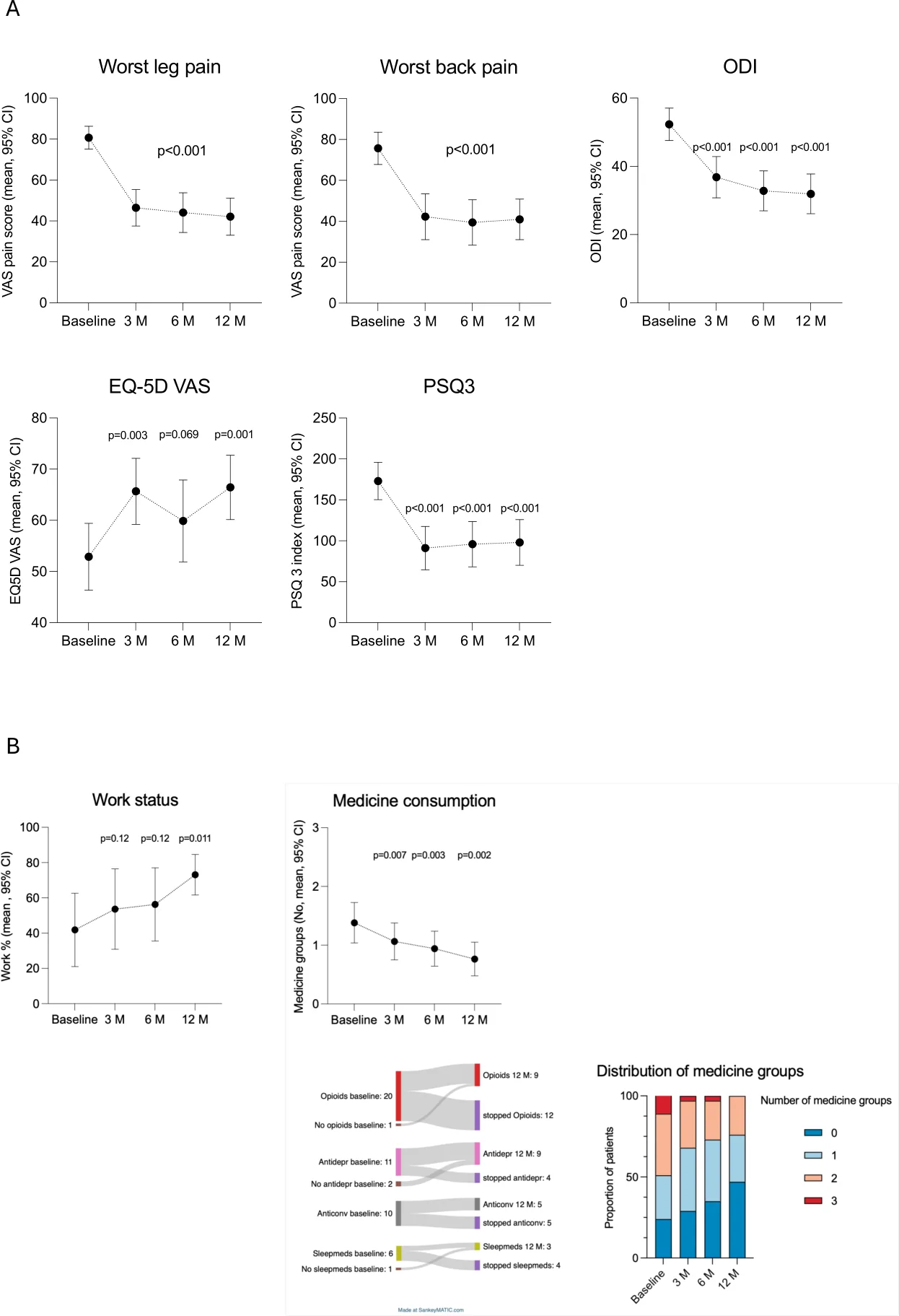
**(A**) Changes between baseline and follow-ups in worst reported leg and back pain VAS, ODI, EQ-VAS, and PSQ-3 (n=34). (B) Change in work status (n=18) and medicine consumption (n=34) during the study period. Medicine consumption is illustrated as a reduction in the mean number of medication groups, along with a graphical representation of this reduction. EQ-VAS, EuroQoL-visual analog scale; PSQ-3, Pain and Sleep Questionnaire Three-Item Index; ODI, Oswestry Disability Index.

PSQ-3 scores improved significantly at all follow-up intervals, with a decrease from 173.1±65.6 at baseline to 98.0±79.9 at 12 months (p<0.001, figure 3A).

#### Work status and medicine consumption

Eighteen patients were able to work (either being in work or on sick leave) at both baseline and 12 months. Among them, the work percentage was significantly increased at the 12 month follow-up (41.8±41.9% vs 73.1±23.2%, p=0.011, figure 3B). A significant reduction was observed in the number of medicine groups used at all follow-ups. From baseline to 12 months, the mean number of medicine groups decreased from 1.38±0.99 to 0.76±0.82, p=0.002. At 12 months, ≥50% of patients had discontinued opioids, anticonvulsants, and sleep medicines (figure 3B).

#### Correlation analyses

From baseline to 12 months, increased mobility correlated significantly with decreased worst leg pain (r=−0.36, p=0.046, figure 4A) and reduced ODI (r=−0.36, p=0.044, figure 4A). Moreover, a significant correlation was found between increased ratio of leg pain compared with back pain at baseline and increased mobility at 12 months (r=0.41, p=0.021, figure 4A, bottom right). No significant correlations were observed between an increase in mobility and changes in other secondary outcomes (figure 4A).

**Figure 4:**
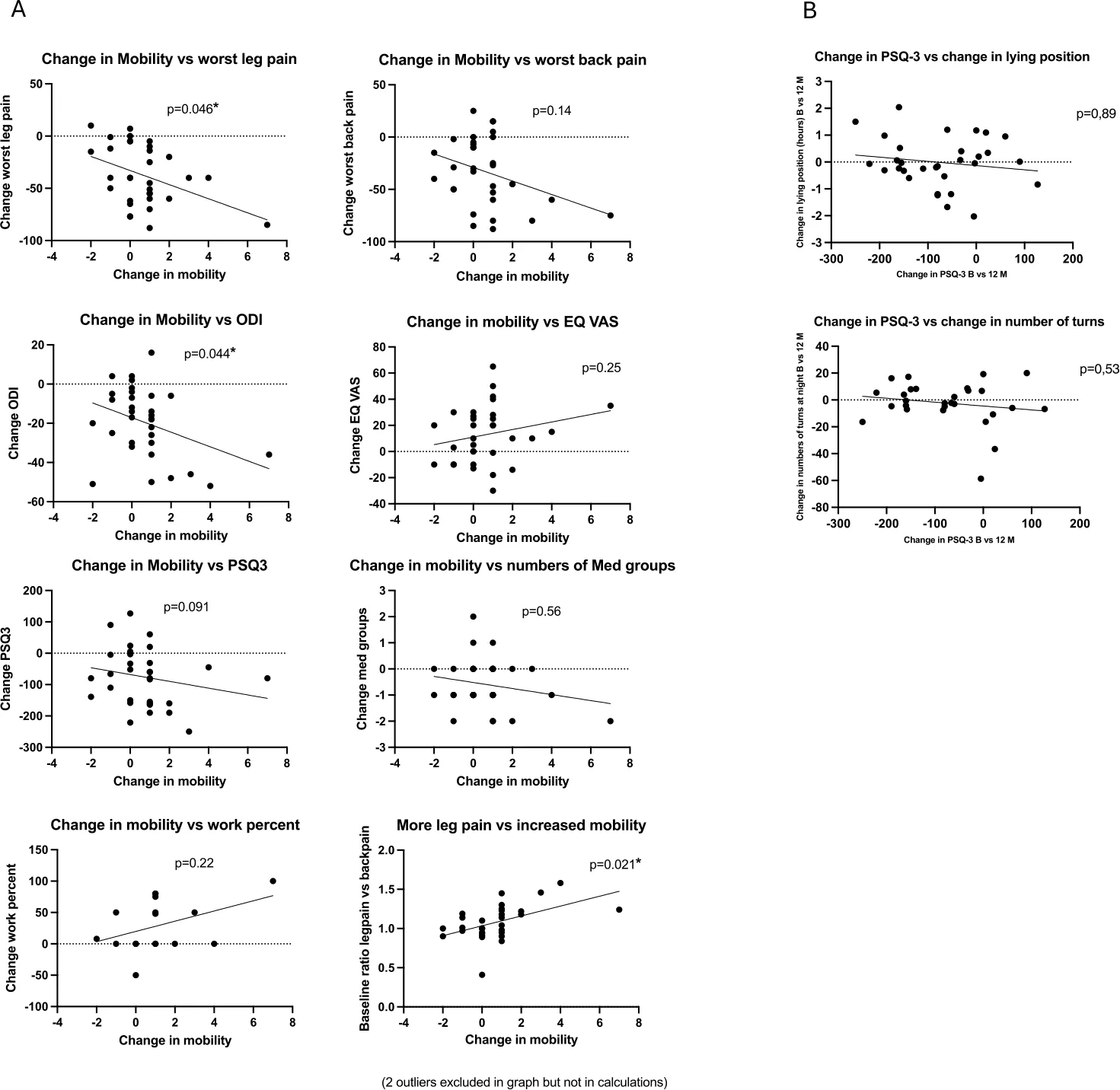
**(A**) Scatter plots depicting correlation analyses between changes in mobility and changes in patient-reported outcome measures (PROMs) from baseline to the 12 month follow-up (n=32). Significant values are denoted with *. (B) Scatter plots illustrating the correlation between changes in PSQ-3 scores and changes in either time spent in the lying position (n=31) or the number of turns during the night (n=28) from baseline to the 12 month follow-up. PSQ-3, Pain and Sleep Questionnaire Three-Item Index.

#### Objective and subjective sleep parameters

Objective data on time spent lying down and number of turns during the night (10 pm–7 am) was accessible for 31 and 28 patients, respectively. No significant changes were observed between baseline and 12 months in either time spent lying down or number of turns during the night (data not shown). Additionally, no significant correlations were found between changes in PSQ-3 scores and changes in either time spent lying down or numbers of turns during the night (figure 4B).

#### Adverse events

Eight patients (16%) experienced a total of eight adverse events. Two patients developed infections during the test trial and were excluded. Three required revision surgery due to lead malfunction (n=2) or lead migration (n=1). One patient developed local pain over the stimulator, requiring reoperation to place it deeper in the subcutaneous tissue. Another patient was treated with antibiotics for a suspected stimulator infection, without requiring explantation. One patient was excluded after unsuccessful revision surgery.

#### Number of leads

No difference was seen in any outcomes irrespective of whether one or two leads were implanted (data not shown).

## Discussion

This study provides new, objective data on the effects of SCS in patients with refractory pain after lumbar spine surgery. Unique features of the study are the continuous 4 week registration of mobility prior to SCS treatment to establish baseline values, and the subsequent registration of mobility up to 12 months of SCS treatment. The results show a significant increase in mobility in patients with predominant leg pain, supporting the efficacy of SCS in this patient category. In addition, medicine consumption significantly decreased, and work capacity significantly increased, demonstrating the societal benefits and cost-effectiveness of SCS treatment in a longer perspective.

Although SCS is generally considered a well-tolerated and useful treatment option for patients with chronic neuropathic pain, the solidity of the evidence regarding treatment efficacy has been questioned.^3 4^ One important concern, in addition to the paucity of robust, long-term, placebo-controlled trials, is the lack of objective outcome measures.^21^ Results are usually based on self-reported questionnaires which are inherently subjective and prone to patient bias. Ideally, assessment of SCS efficacy should also include objective parameters measured over longer periods of time.

A meaningful treatment effect of SCS, in relation to pain reduction, has been suggested to be increased physical activity.^12^ Previous studies have attempted to objectively demonstrate this relationship in patients with chronic pain treated with SCS. In these studies, measurements were either made using external accelerometers with intermittent, short-term recordings for up to 1 week^6 7 22^ or smartwatches measuring heart rate, heart rate variability, step count, and stand time with a high number of missing data.^23 24^ In two studies with data from smartwatches, a correlation was observed between objective measures and various PROMs.^23 24^ In contrast, another study utilizing an implantable accelerometer identified a discrepancy between objective mobility and PROMs, further emphasizing the importance of including objective parameters when reporting outcomes after SCS treatment.^21^

In a recent, small feasibility study, the correlation between objective measurement tools and self-reported questionnaires was investigated in patients with PSPS2 treated with SCS.^9^ The authors used the same stimulator type as in our study to objectively evaluate mobility, but only for 1 week prior to SCS treatment and with a short follow-up of 3 months. Similar to our study, no increase in recorded mobility was seen after 3 months of SCS treatment. They found a correlation between mobility and ODI, without analyzing the correlation between change in mobility and change in ODI.

For the 32 patients in our study with recorded mobility data, mobility increased gradually, reaching statistical significance at 12 months. This indicates a gradual wash-in effect of SCS for improvement of mobility and suggests that it takes time for patients accustomed to inactivity to increase their mobility levels. Interestingly, when analyzing patients with predominant leg or back pain separately, a significant increase in mobility at 12 months was only seen for patients with predominant leg pain, despite similar reduction in VAS for both groups. The mobility levels remained unchanged in patients with predominant back pain. There was a significant correlation between increased mobility and reduction in leg pain that was not observed for back pain. Moreover, a higher degree of leg pain in relation to back pain at baseline correlated with increased mobility at 12 months. The underlying mechanisms behind the difference in mobility changes for patients with predominant leg pain compared with those with predominant back pain are unclear. One possible explanation for the observed difference may be that a reduction of leg pain, which is often neuropathic and radicular in nature, may lead to a more direct improvement in ambulatory function. In contrast, back pain often has a musculoskeletal component, and despite a general perception of pain reduction, patients may remain functionally limited due to movement-related discomfort.

The back pain component in patients with PSPS2 has posed a treatment challenge with SCS. Several new treatment options, in addition to traditional, paresthesia-based, open-loop SCS, have evolved to improve the outcomes. The novel, paresthesia-free HD and DTM SCS modalities used in this study performed better than traditional tonic stimulation in terms of improved mobility. Although there is strong evidence for SCS efficacy based on composite PROM data in patients with PSPS2, more studies, preferably without industry involvement and using various stimulation forms with inclusion of objective, measurable data, are needed to further explore the response to SCS in patients with back pain.

The correlation demonstrated in our study between increased mobility and improvement in ODI indicates that ODI provides an adequate self-assessment of disability in patients with PSPS2. There were no significant correlations between improvement in sleep quality (PSQ-3) and time lying down or number of turns at night. This discrepancy between subjective and objective sleep parameters is in line with previous findings.^25 26^

There are limitations to our study, the main limitation being lack of a control group with PSPS2 patients not treated with SCS. For practical reasons, it was not feasible to include a non-SCS treated group equipped with an external stimulator adhered to the skin for a continuous period of 12 months to register mobility, nor was it feasible out of ethical aspects to implant patients with an SCS system to perform these recordings without activating the stimulation for the 12 months study duration. However, the 4 week baseline recordings of position and mobility prior to any SCS procedure provide a reliable, objective within-subject control, reducing the risk of patient bias. Moreover, utilizing continuous mobility data from an implanted device also reduces possible patient influence in the long term compared with data from wearable accelerometers collected for a limited period of time, or smart watches with a high number of missing datapoints. In this study, mobility is defined by the accelerometer-based parameter “upright mobile,” which was set to capture periods of continuous movement lasting ≥30 s. While mobility should not be directly extrapolated to physical activity, we consider the mobility outcome measure to be a reasonable and relevant proxy for evaluating activity levels in this patient population. To what extent and in what way SCS may change overall physical activity remains, however, to be established.

Another limitation is the absence of an established MCID for the mobility parameter used. Although a statistically significant increase in mobility was observed, it remains unclear whether this change represents a clinically meaningful improvement. There is therefore a need to define MCIDs for mobility measures. Future research should focus on determining such thresholds to enhance the interpretability of objectively recorded mobility data in chronic pain patients.

## Conclusions

This study presents an objective method of long-term assessment of mobility levels in patients treated with SCS. The finding of significantly increased mobility in patients with PSPS2, preferably those with predominant leg pain, supports SCS effectiveness. While mobility is not necessarily equivalent to physical activity, it provides a valuable and quantifiable proxy. Future research is warranted to better characterize the nature and clinical relevance of the mobility improvements associated with SCS treatment. The objective data should be weighed together with patient-reported outcomes when evaluating the overall effects of SCS on chronic pain.

## Supplementary material

10.1136/rapm-2025-106726 online supplemental file 1

## Data Availability

Data are available upon reasonable request.
