# Supplementary material for: Objective, long-term mobility data in patients with chronic pain after lumbar spine surgery treated with spinal cord stimulation—a prospective, multicenter trial
Source: Reg Anesth Pain Med. 2025 Jul 10;51(10):e106726. doi: 10.1136/rapm-2025-106726 (PMC13638309; doi:10.1136/rapm-2025-106726)
Supplement: Supplementary file 1 [file rapm-51-10-s001.pdf]

Ansökan om etikprövning – *Bilaga 1*

Beskrivning av forskningsprojektet

2. Typ av forskning

2.1. På vilket eller vilka sätt handlar projektet om forskning enligt 3-4 §§ etikprövningslagen?

- ☐ 3 § 1 Forskningen kommer att samla in känsliga personuppgifter.
- ☐ 3 § 2 Forskningen kommer att samla in personuppgifter om lagöverträdelser.
- ☒ 4 § 1 Forskningen innebär ett fysiskt ingrepp på en forskningsperson.
- ☐ 4 § 2 Forskningen utförs enligt en metod som syftar till att påverka forskningspersonen fysiskt eller psykiskt, eller så innebär forskningen en uppenbar risk att skada forskningspersonen.
- ☐ 4 § 3 Forskningen avser studier på biologiskt material som har tagits från en levande människa och kan härledas tillbaka till denna människa.
- ☐ 4 § 4 Forskningen avser ett fysiskt ingrepp på en avliden människa.
- ☐ 4 § 5 Forskningen avser studier på biologiskt material som tagits från en avliden människa och kan härledas tillbaka till denna människa.

- ☐ Forskningen faller inte under etikprövningslagens tillämpningsområde.

Forskningsperson: De levande människor som forskningen avser.

Personuppgifter: All slags information som direkt eller indirekt kan hänföras till en fysisk person som är i livet.

Känsliga personuppgifter: Känsliga personuppgifter är uppgifter som avslöjar ras eller etniskt ursprung, politiska åsikter, religiös eller filosofisk övertygelse, medlemskap i fackförening, hälsa, en persons sexualliv eller sexuella läggning, genetiska uppgifter och biometriska uppgifter som entydigt identifierar en person.

Personuppgifter om lagöverträdelser: Personuppgifter om lagöverträdelser som innefattar brott, domar i brottmål, straffprocessuella tvångsmedel eller administrativa frihetsberövanden.

2.2. [Om 3 § 1] **Ange vilken typ av känsliga personuppgifter som kommer behandlas i projektet.**

- ☐ ras eller etniskt ursprung
- ☐ politiska åsikter
- ☐ religiös eller filosofisk övertygelse
- ☐ medlemskap i fackförening
- ☐ hälsa
- ☐ en persons sexualliv eller sexuella läggning
- ☐ genetiska uppgifter
- ☐ biometriska uppgifter som entydigt identifierar en person.

Personuppgifter: All slags information som direkt eller indirekt kan hänföras till en fysisk person som är i livet.

Känsliga personuppgifter: Känsliga personuppgifter är uppgifter som avslöjar ras eller etniskt ursprung, politiska åsikter, religiös eller filosofisk övertygelse, medlemskap i fackförening, hälsa, en persons sexualliv eller sexuella läggning, genetiska uppgifter och biometriska uppgifter som entydigt identifierar en person. Enligt definition i EU:s Dataskyddsförordning.

OBS! Om inga känsliga personuppgifter kommer behandlas i projektet så behöver frågan inte besvaras.

3. Syfte och frågeställningar

3.1. Skriv en populärvetenskaplig sammanfattning av forskningsprojektet (max 300 ord).

Beskriv projektet på ett sammanfattande vis. Tänk på att texten ska kunna förstås av personer som inte har vetenskaplig kompetens. Undvik därför terminologi som kräver specialkunskaper.

Patienter med kronisk nervsmärta kan bli smärtlindrade med ryggmärgsstimulering när konservativa, icke-operativa behandlingar har prövats och inte fungerar. Ryggmärgsstimulering är en etablerad och vetenskapligt väldokumenterad, minimalt invasiv operativ metod där elektroder införs i epiduralrummet i spinalkanalen under lokalbedövning för stimulering av ryggmärgen för att dämpa smärtimpulser. I stimuleringssystemet ingår en pacemaker (strömkälla) som läggs under huden och kopplas till elektroderna om patienten efter en testperiod upplever en adekvat smärtlindring. Stimuleringen styrs via en fjärrkontroll vilken sköts av patienten. Det vanligaste sjukdomstillståndet som svarar väl på ryggmärgsstimulering är kvarstående smärta efter operationer i ländryggen, t ex pga diskbråck.

En svaghet i den vetenskapliga dokumentationen avseende effekterna av ryggmärgsstimulering mot smärta är avsaknaden av objektiva mätmetoder. Utvärderingen har hittills baserats på frågeformulär med registrering av subjektiva utfall, såsom uppfattad smärtlindring och förbättring av sömnkvalitet, vilka kan värderas på olika sätt av patienterna. Den aktuella studiens mål är att utvärdera effekten av ryggmärgsstimulering med en ny generation av stimuleringsutrustning som kontinuerligt registrerar rörelser och kroppslägen och på så sätt kan ge objektiva data i ett långtidsperspektiv om eventuell förbättring av aktivitetsgrad och sömn. Patienter med behandlingsresistent smärta i rygg/ben efter operation i ländryggen, vilket betydligt nedsätter aktivitetsgrad, kommer att inom ramen för den rutinmässiga verksamheten vid neurokirurgiska kliniken, SU/SS opereras med inläggning av en ny typen av pacemaker (Intellis, Medtronic), som kan insamla objektiva data baserat på patienternas rörelsemönster. Studien är ett europeiskt samarbetsprojekt som i ett vidare perspektiv ytterligare kan stärka evidensen för effekten av ryggmärgsstimulering baserat på objektiva effektmått, såsom förbättrad aktivitetgrad, och därmed säkerhetsställa förbättrade utfall. Alla systemkomponenter är marknadssläppta och godkända för klinisk användning inom EU samt används redan på SU/SS. Planen är att data från totalt 120 patienter från 10-12 olika europeiska centra skall samlas in. Studien är beräknad att avslutas 2022.

3.2. Vad är det vetenskapliga syftet med projektet?

Beskriv det övergripande syftet med projektet. Redogör för vad det är för forskningsproblem som projektet ska behandla och vilka avgränsningar som gjorts.

Den vetenskapliga dokumentationen avseende effekterna av ryggmärgsstimulering för behandling av kronisk smärta har hittills baserats på subjektiva patientdata, såsom uppfattad smärtlindring samt förbättrad livskvalitet och sömn. Behovet av kompletterande, objektiva metoder för att utvärdera effekten av behandlingen har varit stort. Det aktuella projektets mål är att utvärdera effekten av ryggmärgsstimulering med en ny typ av stimuleringsutrustning som efter sedvanlig inläggning under huden kontinuerligt registrerar rörelser och kroppslägen och på så sätt kan ge objektiva data i ett långtidsperspektiv om eventuell förbättring av aktivitetsgrad och sömn. Studien kommer att fokusera på patienter med behandlingsresistent smärta i rygg/ben efter operation i ländryggen, vilket betydligt nedsätter rörligheten och den fysiska förmågan. Förutsättningarna för att dokumentera förbättringar i aktivitetsgrad med den nya typen av stimularingsutrustning torde vara lättast att uppfånga hos denna patientgrupp.

3.3. Vilka är de vetenskapliga frågeställningarna?

Ange klart och tydligt den eller de vetenskapliga frågeställningarna i projektet.

Primär frågeställning: Erhålla objektiva effektmått vid långtidsbehandling med ryggmärgsstimulering baserat på registrering av rörelsemönster och aktivitetsgrad med det kommersiellt tillgängliga batteriet Intellis (Medtronic) hos patienter med kronisk, terapiresistent smärta efter ryggoperation.

Sekundär frågeställning: Relatera utfallet vad avser aktivitetsgrad och rörelsemönster under dag- respektive nattid till subjektiva data baserat på standardiserade frågeformulär för skattning av i) smärta (Visual Analogue Scale, VAS), ii) livskvalitet (Euro Qol 5 Dimensions, EQ-5D, iii), sömn (Pittsburg Sleep Quality Index, PSQI), och iv) funktionshinder (Qswestry Disability Index, ODI).

4. Metod

4.1. Redogör för metod inkl. proceduren, tekniken eller behandlingen.

Det ska framgå hur projektet planeras att genomföras. Beskriv insamlade datas karaktär och ange hur datas tillförlitlighet ska säkerställas.

Om enkäter och intervjuer ingår ska tillvägagångssätt, frågornas innehåll och hur slutsatser dras beskriva. Bifoga enkäter och skattningsskalor.

För medicinsk forskning ska anges t.ex. typer av ingrepp, mätmetoder, antal besök, tidsåtgång, doser, och administrationssätt för eventuella läkemedel.

Om projektet är ett samarbete med utlandet ska det tydligt framgå vilken del av forskningen som utförs i Sverige och vilken del av forskningen som utförs utanför Sverige. Det är endast forskning som ska utföras i Sverige som Etikprövningsmyndigheten kan pröva.

Projektet är del i en europeisk multi-centerstudie för att med objektiva mått bedöma effekten av ryggmärgsstimulering. Data avseende aktivitetsgrad och rörelsemönster skall samlas in på patienter som har en ny eller kvarstående, terapiresistent smärta i rygg och/eller ben efter ländryggskirurgi där ytterligare öppen kirurgi inte är aktuell och där patienten bedömts som lämplig för behandling med ryggmärgsstimulering. Data kommer att samlas in från journaler vad avser patientens smärttillstånd innan behandling (upp till 10 år innan första behandling med ryggmärgsstimulering).

Efter en poliklinisk läkarbedömning som tar 30-60 min får patienten muntlig och skriftlig information om studien om hon/han befinns lämpliga för inklusion. Patienten skall ge ett skriftligt samtycke till sitt deltagande i studien. Efter detta initieras vid ett separat läkar/sjuksköterskebesök en kontinuerlig registrering av rörelsemönster och aktivitetsgrad via ett Intellisbatteri som aktiverats för att registrera dessa parametrar och som fästes externt på huden på bålen på de deltagande patienterna med en hudvänlig häfta/tejp. Det som registreras är de fyra liggande positionerna (sidoläge höger och vänster samt rygg- och magläge) samt upprätt läge och upprätt läge i rörelse, således sammanlagt 6 parametrar. Denna kontrollregistrering skall pågå i 4 veckor för att ge basdata om respektive patients rörelsemönster och aktivitetsgrad under dag respektive natt innan ev behandling med ryggmärgsstimulering. I studien ingående patienter erhåller ett ID-nummer som inte är möjligt för utomstående att koppla till patientens identitet.

Efter 4 veckors basregistrering avläses och sammanställs ovanstående data varefter det externa batteriet tas bort. Patienten opereras därefter i en separat seans med inläggning av en epidural elektrod för behandling med ryggmärgsstimulering enligt vedertagna rutiner på Sahlgrenska universitetssjukhuset. Patienten genomgår på sedvanligt sätt ett test på 2-3 veckor för att bestämma om hon/han får adekvat smärtlindring och således är kandidat för inläggning av ett permanent stimuleringssystem med Intellisbatteri. Hos de patienter som svarat med adekvat smärtlindring (>50%) läggs det nya, permanenta Intellisbatteriet under huden på det ställe där det externa batteriet för registrering av basdata var fäst. Det permanenta batteriets dataregistrering aktiveras varefter avläsning av rörelsemönster och aktivitetsgrad görs efter 3, 6 och 12 månader. Jämförelse görs med basdata från 4-veckors registreringen före inläggningen av stimuleringssystemet. Data avseende aktivitetsgrad och rörelsemönster kommer att jämföras med subjektiva parametrar utifrån validerade skalor från standardiserade frågeformulär som patienten lämnar in vid varje uppföljning för bestämning av:
i) Smärta (Visual Analogue Scale, VAS)
ii) Livskvalitet (Euro Qol 5 Dimensions, EQ-5D
iii) Sömn (Pittsburg Sleep Quality Index, PSQI)
iv) Funktionshinder (Qswestry Disability Index, ODI)
Vidare kommer data att insamlas avseende:
- Läkemedelsanvändning
- Användning av sjukhusresurser (t.ex. interventioner, diagnostik)
- Programmeringsinställningar
- Patientents användningsgrad av ryggmärgsstimulatorn
- Data från olika besök (t.ex. utprövning av ryggmärgsstimulering, andel permanenta implantat av pulsgenerator, revisionsbehov efter inläggning av komplett system, omprogrammeringsbehov)
- Hur väl smärtområdet täcks in av stimuleringen

- Eventuella komplikationer som kan relateras till stimuleringssystemet

Alla uppföljande besök för avläsning av data samt eventuell justering för optimering av stimuleringen fram till tidpunkten för studiens avslut beräknas ta 30-60 min.

Ovanstående tillvägagångssätt för selektion av patienter, operationsteknik samt för insamlande och registrering av data är standardiserat och utförs enhetligt i samtliga deltagande länder.

4.2. Redogör för på vilket sätt metoden skiljer sig från klinisk rutin eller den ordinarie behandlingen.

Ange vad som avviker eller tillkommer med anledning av forskningsprojektet.

Det enda som skiljer metoden från de rutiner som används vid behandling med ryggmärgsstimulering vid Sahlgrenska universitetssjukhuset är den preoperativa registreringen av basdata under 4 veckor via det externt placerade Intellisbatteriet som fästes på huden på de deltagande patienterna. Batteriet täcks av plast vilket möjligör för patienterna att duscha och sköta sin hygien som vanligt under de 4 veckor som denna registrering pågår. Den hudvänliga häftan på vilken batteriet är fäst mot huden kan bytas vid behov. Detta moment är avgörande för hela studien då det ger en upplysning om/i vilken utsträckning aktivitetsgraden kan förbättras av ryggmärgsstimulering. Basdata jämförs med data vid uppföljningen 3, 6 och 12 månader efter att behandlingen med ryggmärgsstimulering påbörjats.

4.3. Redogör för tidigare erfarenheter (egna och/eller andras) av den använda proceduren, tekniken eller behandlingen.

Redogör för vilken erfarenhet och kompetens som medverkande forskare har av att använda den procedur, teknik eller behandling om man planerar att använda i projektet. Redogör även för vilken erfarenhet som finns generellt eller globalt.

Ryggmärgsstimulering är en etablerad metod sedan 1980-talet för behandling av patienter med kronisk smärta vid neurokirurgiska klinken, Sahlgrenska universitetsjukhuset. Årligen läggs 60-80 nya stimuleringssystem vid kliniken av undertecknad och medarbetare på indikationen smärta. Totalt genomförs över 200 operationer per år vid kliniken vilka är relaterade till behandling med ryggmärgsstimulering. Metoden är etablerad globalt och anses idag vara rutin vid behandling av främst nervutlöst, terapiresistent smärta. Globalt läggs cirka 50000 stimulatorer per år in på denna indikation.

Metoden att fästa batteriet via en typ av hudskyddsplatta (tvådelsbandage) mot huden för registreringen av basdata är väl utprovad och används rutinmässigt för tarmstomier under längre tidsperioder utan olägenhet för patienterna.

5. Tidsplan

5.1. Förväntat startdatum för projektet:

Ange om möjligt, beräknad start av projektet.

Beräknad start av projektet är under andra kvartalet av 2020

5.2. Förväntat slutdatum för projektet:

Ange om möjligt, beräknat slut av projektet.

Förväntat slutdatum för projektet med komplett dataanalys är 2022

5.3. Tidsplan för de olika delar som ingår i projektet:

Inklusion av patienter (120 st) vid de deltagande europeiska sjukhusen kommer att påbörjas under andra kvartalet av 2020 och förväntas vara slutförd under 2021.

Komplett datainsamling (uppföljning ett år efter inläggning av system för ryggmärgsstimulering) från inkluderade patienter förväntas vara slutförd under 2022 varefter analys av data kommer att påbörjas. Denna analys beräknas vara klar under slutet av 2022.

Sammanställning av data för publikation i refereegranskad tidskrift beräknas ske under första kvartalet 2023. Neurokirurgiska klinken, Sahlgrenska universitetssjukhuset är huvudansvariga för studien.

Redogör för projektets tidsschema, hur de olika delarna förhåller sig till varandra, vid behov kan ett förklarande flödesschema bifogas.

6. Datainsamling

6.1. Redogör för datainsamling och datas karaktär.

Redogör för hur datainsamlingen ska gå till. Beskriv den data som ska samlas in och hur den ska samlas in.

Registrering av data (se under punkt 4.1 vilken data som kommer att samlas in) kommer att ske på formulär (case report forms; CRF). Data från studien kommer samlas in via ett webbaserat verktyg. Inmatning av data kommer att ske via ett säkert nätverk. Metoden att samla in och lagra data sker i ett dataregistreringssystem enligt 21 CRF Part 11. Mjukvaran och databasen har tillverkats i enlighet med legala och regulatoriska krav på dess användning inom ett validerat system för att bedriva kliniska studier med hjälp av elektronisk data och signaturer.

6.2. Redogör för det statistiska underlaget för studiepopulationen/ undersökningsmaterialets storlek.

Redogör för de beräkningar och överväganden som gjorts för att komma fram till hur många deltagare som behövs för att uppnå tillräcklig statistisk styrka. Redogörelsen av hur man kommit fram till lämpligt urval ska beskriva projektets möjligheter att besvara frågeställningarna.

Antalet patienter per center som kommer att analyseras är begränsat till 12 st. Totalt kommer 120 patienter att inkluderas utifrån en powerberäkning att med 80% sannolikhet kunna påvisa en förväntad effekt (smärtlindring) på 50%. Data utifrån de primära och sekundära frågeställningarna kommer att analyseras med ett två-sidigt 5% konfidensintervall och jämföras med baseline (data innan patienten fick behandling med ryggmärgsstimulering). Alla patienter som uppfyller inklusionkriterierna kommer att inkluderas i följdordning för att minimera bias i urvalet. Frågeställningarna kommer att bli analyserade med avseende på korrelation mellan kliniska faktorer före och efter operationen med inläggning av stimuleringssystemet. Detta förfarande inkluderar potentiella covarians prediktorer av post-operativ effekt. Interimanalyser kommer inte att göras under studiens gång.

6.3. Hur kommer undersökningsprocedurerna att dokumenteras?

Redogör för hur undersökningarna och eventuella ingrepp dokumenteras. Ange om band- och videoinspelningar kommer användas.

Undersökningsprocedurer, operativa ingrepp, vårdtillfällen och polikliniska besök dokumenteras i patientjournal vid Sahlgrenska universitetssjukhuset som förs enligt patientdatalagen, på samma sätt som i vanlig sjukvård. Inga band- eller videoinspelningar kommer att användas.

6.4. Hur kommer insamlad data att hanteras och förvaras?

Redogör för hur data kommer att hanteras efter insamlingen. Om data kommer att pseudonymiseras (”kodas”) ska kodningsförfarandet beskrivas. Det ska framgå var kodlistorna/kodnycklarna kommer att förvaras samt vem/vilka som kommer ha tillgång till dem. Det ska även framgå hur länge data kommer att sparas samt om det kommer att avidentifieras (genom att kodnyckel förstörs) eller förstöras i sin helhet.

All data kodas och det upprättas en kodnyckel som förvaras på ett säkert ställe så att ingen obehörig kan få tillgång till forskningsdata. All data kommer att ha spårbarhet så att vem som lagt in data och när framgår, s.k. audit-trail. Alla personrelaterade uppgifter ersätts med en individuell och studiespecifik kod (patientnummer). Patientidentifikationsformulär som länkar ihop patientnummer med personuppgifter för varje patient upprättas och förvaras på varje center i en prövarpärm som förvaras på säkert ställe utan åtkomst för obehöriga. Back-up på databasen kommer att göras regelbundet. Forskaren signerar CRF:er elektroniskt. Förändringar i redan registrerade data kräver en ny signatur från forskaren för att bekräfta och godkänna ändringarna. Alla ändringar av kliniska data som görs kommer att vara sparade elektroniskt och kommer kunna följas av huvudansvarig för studien.

Prövarpärm och kopia på elektroniska CRF sparas 10 år efter avslutad studie varefter materialet avidentifieras och förstörs i sin helhet. Varje studiecenter kommer att få en CD-ROM skiva eller tillgång till all CRF-data för respektive centers patienter. Ett källdatadokument kommer att upprättas där det framgår var källdata för rapporterade uppgifter i studien finns dokumenterad.

7. Etiska överväganden

7.1. Vilka risker kan ett deltagande medföra för de forskningspersoner som ingår i forskningsprojektet?

Risker kan vara av olika karaktär. Det kan exempelvis vara fysisk eller psykisk skada, smärta, obehag, integritetsintrång på kort eller lång sikt. Ange vilka risker det kan finnas för de forskningspersoner som deltar i detta forskningsprojekt.

Medverkan i den här studien medför inga ytterligare risker utöver de som förekommer vid rutinmässig behandling med ryggmärgsstimulering hos patienter utanför studien. Allvarliga komplikationer i samband med ryggmärgsstimulering är ytterst sällsynta, de flesta komplikationer (främst infektion runt batteriet efter inläggning under huden och ändrat elektrodläge) är lätt åtgärdbara och leder inte till några nackdelar eller men för patienten i ett långtidsperspektiv. Det som de inkluderade patienterna eventuellt kan uppleva är irritation från hudskyddsplattan (tvådelsbandage) som används för att fästa Intellisbatteriet mot huden för registreringen av basdata innan testperioden med ryggmärgsstimulering. Denna typ av platta är dock väl utprovad och används rutinmässigt för tarmstomier under längre tidsperioder utan olägenhet för patienterna.

Eventuella komplikationer registreras i patientjournalen för att kunna sammanställas som del i studiens resultat samt i fortlöpande kvalitetssäkringssyfte. De i studien ingående patienterna utsätts inte för några extra ingrepp utöver de som normalt förekommer hos patienter som behandlas med ryggmärgsstimulering. Deltagande i den här studien kommer att sekretessbeläggas så som det krävs i de tillämpliga lagarna. Patienterna har rätt till full integritet och all information som samlas in i denna studie är konfidentiell enligt lag (Personuppgiftslagen 1998:204). Några ökade risker för integritetsintrång finns ej jämfört med patienter som behandlas med ryggmärgsstimulering utanför studiens ram.

7.2. Vilken nytta kan ett deltagande medföra för de forskningspersoner som ingår i forskningsprojektet?

Ange vilken hjälp de forskningspersoner som deltar i detta forskningsprojekt kan få som ett resultat av forskningsprojektet.

Någon förutsägbar nytta eller fördelar för deltagande forskningspersoner i ett längre perspektiv finns inte, förutom den som behandlingen i sig själv kan innebära vad avser smärtlindring, förbättrad funktionsgrad och livskvalitet, minskad medicinförbrukning samt återgång till arbete.

7.3. Gör en värdering av förhållandet mellan riskerna och nyttan av projektet.

Forskning får enligt etikprövningslagen bara godkännas om riskerna som den kan medföra för forskningspersonernas hälsa, säkerhet och personliga integritet uppvägs av dess vetenskapliga värde.

Inga risker för patienterna eller etiska problem kan identifieras i ett vidare perspektiv inom ramen för det aktuella projektet. Behandlingen och riskerna med ryggmärgsstimulering (se punkt 7.1.) för de i studien inkluderade patienterna skiljer sig inte från de rutiner för ryggmärgsstimulering som normalt gäller för patienter utanför studien. Vi hoppas att resultaten skall bidra till bättre kunskap om att med objektiva mått kunna belysa nyttan med ryggmärgsstimulering vid behandling av patienter med långvarig smärta.

7.4. Beskriv hur projektet har utformats för att minimera riskerna för forskningspersonerna.

Forskning får enligt etikprövningslagen bara godkännas om det förväntade resultatet inte kan nås på ett annat sätt som innebär mindre risker för forskningspersoners hälsa, säkerhet och personliga integritet.

Behandling av personuppgifter som avses i 3 § får godkännas bara om den är nödvändig för att forskningen ska kunna utföras.

Observera att om en behandling ska prövas för första gången på människa måste detta tydligt framgå och relevanta säkerhetsrutiner tydligt beskrivas.

Ange vilka risker som ett deltagande i forskningsprojektet kan innebära. Finns det risker t.ex. med att ingå i en kontrollgrupp?

Förutom basregistreringen under 4 veckor av rörelsemönster och aktivitetsgrad skiljer sig ej den erbjudna behandlingen för de i studien ingående patienterna från den behandling som erbjuds patienter utanför studien vid Sahlgrenska universitetssjukhuset. Någon kontrollgrupp ingår ej i studien eftersom de inkluderade patienterna är sina egna kontroller.

7.5. Identifiera och precisera om eventuella etiska problem (nackdelar/fördelar) kan uppstå i ett vidare perspektiv genom forskningsprojektet.

Redovisa t.ex. om vissa grupper (andra än de forskningspersoner som ingår i forskningsprojektet) kan komma att utpekas respektive få hjälp som ett resultat av projektet. Frågan avser även de indirekta riskerna eller den indirekta nyttan. T.ex. genetisk påverkan på kommande generationer eller om resultaten på annat sätt kan tänkas skada vissa grupper.

Inga eventuella etiska problem kan identifieras i ett vidare perspektiv genom forskningsprojektet (se punkt 7.1-7.4).

8. Forskningspersoner

8.1. Hur görs urvalet av forskningspersoner?

Forskningsperson: De levande människor som forskningen avser.

Beskriv vilka forskningspersoner som kommer inkluderas i projektet. Redogör för de överväganden som gjorts vid valet av forskningspersoner. Om vissa grupper utesluts från deltagande i projektet ska en motivering till uteslutandet framgå.

Redogör även för hur forskaren kommer i kontakt med eller får kännedom om lämpliga forskningspersoner.

Studien kommer att inkludera patienter som har en ny eller kvarstående, terapiresistent smärta i rygg och/eller ben efter ländryggskirurgi där konservativ medikamentell och fysikalisk behandling ej haft avsedd effekt och ytterligare öppen kirurgi inte är aktuell. Patienter med behandlingsresistent smärta i rygg/ben efter operation i ländryggen uppvisar en betydande nedsättning vad avser rörlighet och fysisk aktivitetsgrad. Förutsättningarna för att dokumentera förbättringar i aktivitetsgrad utifrån en adekvat smärtlindring med den nya typen av stimuleringsutrustning torde vara lättast att uppfånga hos denna patientgrupp.

Patienterna har remitterats till neurokirurgiska kliniken för specifik bedömning under frågeställningen lämplighet för ryggmärgsstimulering från annan mottagning, oftast ortoped- eller smärtmottagning där konservativa och kirurgiska behandlingsalternativ ansetts vara uttömda efter en multidisciplinär bedömning. Efter ett besök vid neurokirurgiska mottagningen med föregående granskning av kliniska uppgifter vad avser patientens sjukhistoria samt aktuellt status görs en sammanvägd bedömning vad avser lämplighet för inklusion i studien.

8.2. Hur många forskningspersoner kommer att inkluderas i forskningsprojektet?

Ange hur många forskningspersoner som totalt kommer inkluderas i projektet samt i förekommande fall hur många som kommer inkluderas i olika delprojekt.

Totalt kommer 120 patienter att inkluderas i studien varav maximalt 12 st från neurokirurgiska kliniken, Sahlgrenska universitetssjukhuset (se punkt 6.2.).

8.3. Vilka urvalskriterier kommer att användas för inklusion?

Ange vilka kriterier som måste uppfyllas för att en forskningsperson ska inkluderas i projektet.

- Terapiresistent kronisk smärta i rygg och/eller ben som har varat minst 6 månader efter det senaste ryggkirurgiska ingreppet. Patienten har bedömts som ej aktuell för ytterligare ryggkirurgi.
- Patientålder 18-70 år.
- Genomsnittlig upplevd smärtintensitet enligt VAS-skalan på 5 eller högre.
- Patienten skall ha informerats muntligt och skriftligt om studien och gett informerat skriftligt samtycke till deltagande.

8.4. Vilka urvalskriterier kommer att användas för exklusion?

Ange vilka kriterier som måste uppfyllas för att en forskningsperson ska exkluderas ur projektet.

- Patienten förväntas ej förstå eller kunna sköta stimuleringsutrustningen.
- Pågånde koagulationsstörning.
- Pågående missbruk av alkohol, narkotika eller receptbelagda opioider.
- Aktiv psykiatrisk sjukdom.
- Aktiv malignitet.
- Tillstånd med ökad generell infektionskänslighet, t ex känd immunbrist.
- Förväntad livslängd <1 år.
- Pågående lokal infektion eller annan sjukdom i huden där man planerar att lägga in batteriet.
- Graviditet.
- Deltagande i annan studie.

8.5. Ange relationen mellan forskare och forskningspersonerna.

Ange forskarens roll i relation till forskningspersonens roll. Det kan t.ex. vara som behandlare (läkare, psykolog, fysioterapeut etc.) och patient/klient, som lärare och student eller arbetsgivare och anställd. All form av relation som kan tänkas medföra risk för påverkan ska beskrivas. Enligt etikprövningslagen ska information och samtycke ägnas särskild uppmärksamhet om forskningspersonen står i ett beroendeförhållande till huvudmannen, forskaren eller antas ha svårigheter att ta tillvara sin rätt.

Forskaren är forskningspersonens (patientens) behandlande läkare där behandlingen utförs enligt de rutiner som används vid neurokirurgiska kliniken, Sahlgrenska universitetssjukhuset för den undersökta patientkategorin. Någon relation som kan tänkas medföra risk för påverkan föreligger inte.

8.6. Vilket försäkringsskydd finns för de forskningspersoner som deltar i forskningsprojektet?

Forskningshuvudmannen har ansvar för att kontrollera att det finns försäkring som täcker eventuella skador som kan uppkomma i samband med forskningen. Ange vilka försäkringar som forskningspersonen kommer att omfattas av samt vilket skydd försäkringarna ger forskningspersonen.

I studien ingående patienter omfattas av den allmänna patientförsäkringen.

8.7. Redogör för den beredskap som finns för att hantera oväntade bifynd eller händelser under forskningsprocessen som kan äventyra forskningspersonernas säkerhet.

Beskriv vilken tillgång forskningsprojektet har till utrustning, personal och kompetens för att hantera eventuellt oväntade komplikationer eller bifynd. Beskriv även vilken planering som finns för att hantera eventuella oväntade komplikationer eller bifynd.

Studien genomförs inom ramen för den offentligt finansierade sjukvården vid Sahlgrenska universitetssjukhuset där all tillgång finns till utrustning, personal och kompetens för att hantera eventuella komplikationer eller bifynd så snart dessa uppstår/uptäcks.

8.8. Kommer ekonomisk ersättning eller andra förmåner betalas ut till forskningspersonerna?

Forskningspersonerna kan utöver ersättning för resor, förlorad arbetsinkomst eller andra utgifter erhålla viss ersättning för obehag och besvär. Ersättningen ska vara skälig. Om barn eller ungdomar under 18 år deltar i forskningsprojektet får sådan ersättning inte vara stor och den bör inte erbjudas i samband med rekryteringen. Vid klinisk läkemedelsprövning med barn eller ungdomar under 18 år får inga incitament eller ekonomiska förmåner ges, undantaget kostnadsersättningar.

Nej

8.8.1. [Om Ja 8.8] **Vilken ekonomisk ersättning kommer betalas ut och när?**

N/A

9. Information och samtycke

9.1. Kommer forskningspersonerna att informeras om forskningsprojektet och tillfrågas om de vill vara med eller inte?

Grundregeln, enligt etikprövningslagen, är att forskning bara utförs om forskningspersonen har informerats och samtyckt till deltagande. Information till forskningspersonerna kan ges både muntligt och skriftligt.

Ja

9.1.1. [Om Ja 9.1] **Hur, när (i vilket skede) och av vem informeras och tillfrågas forskningspersonerna?**

Beskriv proceduren för hur information ges och samtycke inhämtas. Vem som frågar, när detta sker och hur samtycket dokumenteras. På vilket sätt säkerställs att forskningspersonen ges betänketid och möjlighet att ställa frågor.

Utförlig redovisning är särskilt viktig när det ingår barn eller personer med nedsatt beslutskompetens i forskningsprojektet.

Studien kommer följa den europeiska GDRP-lagstiftningen. Tilltänkta forskningspersoner erhåller muntlig och skriftlig information tillsammans med samtyckesformulär om de befunnits vara lämpliga kandidater för inklusion i studien i samband med poliklinisk läkarbedömning vid neurokirurgiuska kliniken, Sahlgrenska universitetssjukhuset. Underskrivet informerat samtycke kommer att inhämtas från de patienter som väljer att delta i studien. De har då möjlighet att ställa frågor och diskutera studien. Information och inhämtande av patientens samtycke sker innan någon studierelaterad

undersökning/behandling påbörjas. Samtycket dokumenteras i patientjournal. Ett exemplar av patientinformationen och det underskrivna samtyckesformuläret behåller patienten och ett exemplar arkiveras i studiepärmen på kliniken.

9.1.2. [Om Nej 9.1] **Motivera varför forskningspersonerna inte ska informeras och tillfrågas.**

Lämna en utförlig redogörelse för de avvägningar som har gjorts och de skäl som ligger till grund för bedömningen att forskningspersonerna inte ska informeras och tillfrågas. Observera att forskning utan information och samtycke endast är möjlig i undantag fall och i den forskning sam avses i 3 § etikprövningslagen, eller 20–22 § i etikprövningslagen.

N/A

9.2. Kommer barn under 18 år att ingå i forskningsprojektet?

För barn under 15 år måste barnets samtliga vårdnadshavare ge sin tillåtelse (samtycka) till att barnet får delta i forskningsprojektet.

Barn mellan 15 och 18 år, som inser vad forskningen innebär för hans eller hennes del, ska informeras om och lämna eget samtycke till forskningen. I andra fall ska barnets samtliga vårdnadshavare ge sin tillåtelse (samtycka) till att barnet får delta i forskningsprojektet.

Forskningen får, trots vårdnadshavarnas tillåtelse (samtycke) inte utföras om barnet motsätter sig att forskningen utförs.

Nej

9.2.1. [Om Ja 9.2] **Ange barnens ålder.**

Max 500 tecken

9.3. Kommer forskningspersoner, vars mening på grund av sjukdom, psykisk störning, försvagat hälsotillstånd eller något annat liknande förhållande inte kan inhämtas, att ingå i forskningsprojektet?

Ange om forskningsprojektet kommer involvera personer som själva inte kan samtycka till sitt eget deltagande p.g.a. sjukdom, psykisk störning, försvagat hälsotillstånd eller liknande tillstånd.

Nej

9.3.1. [Om Ja 9.3] **Motivera varför denna grupp av forskningspersoner ska ingå i projektet.**

Forskning utan samtycke på denna grupp av forskningspersoner får endast utföras om forskningen förväntas ge en kunskap som inte är möjlig att få genom forskning med samtycke.

Forskningen ska dessutom förväntas leda till direkt nytta för forskningspersonen. Alternativt ska forskningen bidra till ett resultat som kan vara till nytta för forskningspersonen eller annan som lider av samma eller liknande sjukdom eller tillstånd samt innebära en obetydlig risk för skada eller obehag för forskningspersonen. Forskningspersonen ska så långt som möjligt informeras personligen om forskningen.

Samråd ska ske med närmaste anhörig. Samråd ska också ske med god man eller förvaltare om frågan ingår i uppdraget som god man eller förvaltare. Forskningen får inte utföras om forskningspersonen i någon form ger uttryck för att inte vilja delta eller om någon av dem som samråd har skett med motsätter sig införandet.

N/A

9.3.2. [Om Ja 9.3] **Beskriv hur samråd med närmaste anhörig, god man eller förvaltare kommer att ske.**

Vid forskning på forskningspersoner, vars mening inte går att inhämta, ska samråd ske med närmaste anhörig som ska få möjlighet att motsätta sig deltagandet. Samråd ska ske med god man eller förvaltare om frågan ingår i uppdraget som god man eller förvaltare.

N/A

10. Registeruppgifter

10.1. Kommer projektet att begära ut uppgifter från ett befintligt register?

Här avses alla typer av register som innehåller personuppgifter eller uppgifter som tidigare varit personuppgifter men senare avidentifierats.

Nej

10.1.1. [Om Ja 9.1] Ur vilket eller vilka register kommer uppgifterna att begäras?

Namnge registret eller registren som uppgifterna kommer att begäras ifrån. Ange även huvudman för respektive register.

N/A

10.1.2. [Om Ja 9.1] Vilka uppgifter kommer att begäras ut och varför?

Beskriv vilken typ av uppgifter som kommer att begäras ut och varför de behövs för att besvara projektets frågeställningar.

En komplett variabellista kan med fördel bifogas som bilaga. Etikprövningsmyndigheten behöver inte alltid en redovisning på variabelnivå för att göra sin bedömning, men det kan ibland underlätta vid begäran hos registerhållaren om den kompletta variabellistan funnits med vid etikprövningen.

N/A

11. Resultat från djurförsök

11.1. Finns det relevanta resultat från djurförsök?

Frågan avser framförallt klinisk behandlingsforskning. Om djurförsök inte har utförts, ange då anledningen till detta.

Ej Aktuellt

11.1.1. [Om Ja 11.1] Redogör för resultaten av djurförsöken

Redogör övergripande för de djurförsök som utförts och vilka resultat de gav. Framförallt ska de resultat som är av relevans för detta forskningsprojekt redogöras för.

N/A

12. REDOVISNING AV RESULTAT

12.1. Hur garanteras tillgång till data för forskningshuvudmannen och medverkande forskare?

Normalt ska den som ansvarar för genomförandet av forskningen ha full tillgång till data. Om flera forskare samverkar i uppdragsforskning bör den forskare som är huvudansvarig för genomförandet i förväg komma överens med övriga forskare om tillgång till data.

Data från studien kommer samlas in via ett godkänt webbaserat verktyg (Research Electronic Data Capture, REDCap) vilket alla i studien ingående forskare har tillgång till. Inmatning av data kommer att ske via ett säkert nätverk. Back-up på databasen kommer att göras regelbundet. Respektive forskare signerar CRF:er elektroniskt. Förändringar i redan registrerade data kräver en ny signatur från forskaren för att bekräfta och godkänna ändringarna.
Alla ändringar av kliniska data som görs kommer att vara sparade elektroniskt och kommer kunna följas av huvudansvarig för studien, som kommer att ha full tillgång till all data via REDCap enligt överenskommelse med övriga forskare. Varje studiecenter kommer att få en CD-ROM skiva eller tillgång till all CRF-data för respektive centers patienter. Ett källdatadokument kommer att upprättas där det framgår var källdata för rapporterade uppgifter i studien finns dokumenterad.

12.2. Vem eller vilka ansvarar för databearbetning och skriftlig redovisning av resultaten?

Ange vem eller vilka som kommer bearbeta och analysera forskningsdata och vem eller vilka som kommer utforma den skriftliga redovisningen. Normalt ska den som ansvarar för genomförandet av forskningen ha full tillgång till data.

Undertecknad, som är huvudansvarig för studien, ansvarar för databearbetning och rapportskrivning tillsammans med ansvarig forskare vid respektive deltagande klinik. Huvudansvarig har full tillgång till all data. Samtliga medverkande forskare får tillgång till resultatet från utvärderingen.

12.3. Hur och när planeras resultaten att offentliggöras?

Ange i vilken form resultaten planeras att offentliggöras. Exempelvis vetenskaplig publicering med peer review, open acess, intern rapport. Ange om möjligt en tidsplan för offentliggörandet.

Studien kommer att publiceras som refereegranskad artikel i internationell vetenskaplig tidskrift när slutligt resultat finnns tillgängligt. En sammanfattning av denna rapport kommer att sändas till involverade forskare och etikprövningsnämnden inom ett år från studiens avslutande. Publikation av resultaten är beräknad till första delen av 2023.

12.4. På vilket sätt garanteras forskningspersonernas rätt till integritet när materialet offentliggörs?

Redogör för hur data presenteras när den offentliggörs och hur forskningspersonernas integritet skyddas vid offentliggörandet.

Resultaten kommer att redovisas endast på statistisk gruppnivå. Patienterna kommer att vara avidentifierade i publikationer. Deltagande i den här studien kommer att sekretessbeläggas så som det krävs i de tillämpliga lagarna. Patienterna har rätt till full integritet och all information som samlas in i denna studie är konfidentiell enligt lag (Personuppgiftslagen 1998:204). Några ökade risker för integritetsintrång kan ej ses jämfört med patienter som behandlas med ryggmärgsstimulering utanför studiens ram.

13. EKONOMISKA FÖRHÅLLANDEN

13.1. Redovisa eventuella ekonomiska överenskommelser med bidragsgivare eller andra finansiärer (namn och belopp).

Redovisa alla överenskommelser om finansiering som har slutits med den eller de som ska genomföra forskningen. Ange vilka belopp som kommer att erhållas för forskningsprojektet och vad ersättningen ska täcka. Ange även eventuella belopp per forskningsperson.

Några överenskommelser om finnansiering finns ej inom ramen för studien då det ej finns några bidragsgivare eller finansiärer.

13.2. Redovisa forskningshuvudmannens, huvudansvarig forskares och medverkande forskares egna ekonomiska intressen.

Redovisa egna ekonomiska intressen i form av t.ex. aktieinnehav, anställning, konsultuppdrag i finansierade företag, eget företag som kan få direkt eller indirekt vinst av forskningen.

Varken huvudansvarig eller medverkande forskare har några ekonomiska intressen relaterat till studien.
